# Mixed logistic regression in genome-wide association studies

**DOI:** 10.1186/s12859-020-03862-2

**Published:** 2020-11-23

**Authors:** Jacqueline Milet, David Courtin, André Garcia, Hervé Perdry

**Affiliations:** 1 Université de Paris, MERIT, IRD, 75006 Paris, France; 2 Université Paris-Saclay, UVSQ, Inserm, CESP, 94807 Villejuif, France

**Keywords:** GWAS, Mixed-models, Logistic regression

## Abstract

**Background:**

Mixed linear models (MLM) have been widely used to account for population structure in case-control genome-wide association studies, the status being analyzed as a quantitative phenotype. Chen et al. proved in 2016 that this method is inappropriate in some situations and proposed GMMAT, a score test for the mixed logistic regression (MLR). However, this test does not produces an estimation of the variants’ effects. We propose two computationally efficient methods to estimate the variants’ effects. Their properties and those of other methods (MLM, logistic regression) are evaluated using both simulated and real genomic data from a recent GWAS in two geographically close population in West Africa.

**Results:**

We show that, when the disease prevalence differs between population strata, MLM is inappropriate to analyze binary traits. MLR performs the best in all circumstances. The variants’ effects are well evaluated by our methods, with a moderate bias when the effect sizes are large. Additionally, we propose a stratified QQ-plot, enhancing the diagnosis of *p* values inflation or deflation when population strata are not clearly identified in the sample.

**Conclusion:**

The two proposed methods are implemented in the R package **milorGWAS** available on the CRAN. Both methods scale up to at least 10,000 individuals. The same computational strategies could be applied to other models (e.g. mixed Cox model for survival analysis).

## Background

Population stratification has long been known to be at the origin of spurious associations in genetic association studies [1]: if the frequency of the phenotype of interest varies across the population strata, it will be associated to any allele the frequency of which varies accordingly. An early and elegant solution to this issue has been the use of family data, notably in the Transmission Disequilibrium Test (TDT) [2] and in the Family Based Association Test (FBAT) [3]. However, these methods imposed the ascertainment and genotyping of affected individuals’ relatives, impairing their practical feasibility. The advent of Genome-Wide Association Studies (GWAS), demanding increasingly large samples to detect weaker and weaker effects, made the problem even more accurate.

Methods adapted to large scale population studies have thus been proposed, among which the Genomic Control [4] and Structured Association methods [5, 6]. The Genomic Control uses the empirical distribution of the genome-wide chi-square statistics to correct the statistic inflation attributable to population structure whereas Structured Association methods infer population strata from genome-wide data before testing for association conditional to the strata.

A major breakthrough was achieved in 2006 with EIGENSTRAT [7], also known as Principal Component Regression (PCR). This conceptually simple but extremely efficient method consists in incorporating the top Principal Components (PC) of the genotype data in a linear model. The next major advance was the introduction of mixed models, which can be interpreted as a generalization of PCR which incorporates all PCs in the model with random effects [8, 9]. Incorporating a few PCs with fixed effects as well in the mixed model might still prove useful to correct statistic inflation at SNPs with large allelic frequency variations across strata [8, 10]. Fast approximate [11] or exact [12] methods for genome-wide analysis of quantitative traits with mixed linear models (MLM) were soon made available.

The analogue of MLM for case-control studies is the mixed logistic regression (MLR). However, fitting the MLR with the Penalized Quasi-Likelihood (PQL) [13] is computationally heavy. Thus, in case-control studies the status was often coded as quantitative trait (0 for control subjects and 1 for disease subjects), and analyzed as such. Nevertheless, Chen et al. [14] proved that when disease prevalence was heterogeneous between populations strata, while the overall distribution of *p* values is well corrected by this method, it leads to conservatives *p* values for some SNPs and to anti-conservative *p* values for others. This behavior was made evident by the mean of quantile-quantile plots (QQ-plots) in which SNPs were categorized according to their allele frequencies in the different population strata. In order to address this issue, Chen et al. proposed GMMAT, a score test for the MLR, which is feasible in GWAS. The *p* values obtained with this test were shown to be well distributed in all SNP categories.

While the score test has a reduced computational burden, its drawback is the absence of an estimation of the variants’ effects. When only the effects of genome-wide significant SNPs are needed, an obvious solution is to fit MLR models including each of these SNPs. When the SNPs’ effects are needed for the whole genome, e.g. for meta-analysis purposes, it is desirable to have a computationally efficient method to estimate these effects. We propose two such methods in this paper.

The strategy to reduce the computational burden is to fit the MLR only once for a “null model”. This null model may incorporate relevant covariates, but no SNP. The SNPs are then tested one by one. Roughly speaking, this is done by using an appropriate method to confront a vector of genotypes with the residues of the null model. This stragegy is shared by the score test (GMMAT) and by the two proposed methods; but while the score test provides association *p* values with no estimate of the variant’s effects, the methods proposed here give such estimates.

One of the proposed method, named hereafter Approximate Maximum Likelihood Estimate (AMLE), is based on a first-order approximation of the MLR, which leads to an approximation of the SNPs effect. The association is tested by a Wald test, which is identical to the score test of [14]. A similar approach has been previously used for mixed linear models [15] and has been recently adapted in SAIGE [15] for MLR, but without an evaluation of its capacity to properly estimate SNP effects.

The other method, named the Offset method, which bears similarities with the methods of [11], consists of first estimating individual effects in a mixed logistic regression model, and then incorporating these effects as an offset in a (non-mixed) logistic regression model.

In the sequel, we evaluate the capacity of logistic regression (LR), MLM, and MLR (using either the score test or the methods mentioned above) to properly take into account population structure associated with heterogeneous prevalence. While Chen et al. were interested in geographically distant populations, spanning several countries in South America, part of our work focuses two geographically very close populations in West Africa, using real genotype data from a recent GWAS [16]. We also use data simulated with a coalescent model [17], reproducing the simulations presented in [14]. We use similar simulations to evaluate the ability of the PQL and of our two methods to properly evaluate SNPs’ effects.

Additionally, we propose to generalize the categorization of SNPs in QQ-plots presented in [14] to variables other than the population strata, including continuous variables as for example the first PC. The interest of this generalization is demonstrated using the same simulations.

All methods are implemented in the R package milorGWAS (for mixed logistic regression in GWAS), freely available on the Comprehensive R Archive Network (CRAN).

**Fig. 1:**
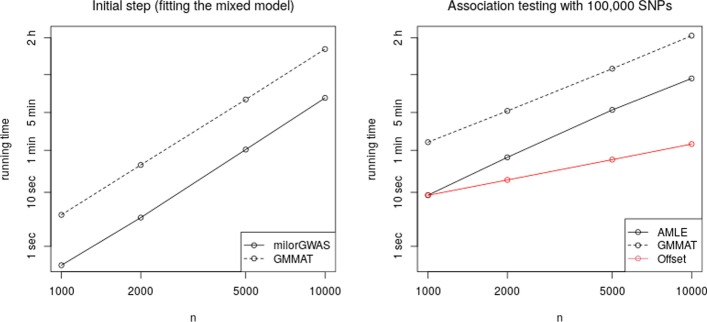
Running times for sample sizes varying from $n = 1000$ to $n=10{,}000$. All computations were run on a Intel i7 at 3.60 GHz with 8 MB of cache memory. Due to the log–log scale, the timing of an algorithm with complexity $O(n^\alpha )$ appears as a straightline with slope $\alpha$: slope 3 for the initial step, 2 for AMLE and GMMAT, and 1 for the Offset method

## Results

### Computational efficiency of the methods

We measured the running times of GMMAT and of the methods AMLE and Offset, implemented in our package milorGWAS, for a sample size *n* varying from 1000 to 10, 000. The results are reported in Fig. 1. The figure is on log–log scale, showing that the results are in good accordance with the asymptotic complexity of the methods, given in the Methods section: the time taken by the initial step (fitting the mixed model) growing as $n^3$, it appears linear on log–log scale with a slope close to 3. Similarly, the complexity $O(n^2)$ of GMMAT and AMLE result in straight lines with a slope of 2. GMMAT is consistently slower than milorGWAS: milorGWAS runs roughly 8 times faster for the initial step, and 6 times faster for the testing step. Part of the difference in the testing step is due to the fact that GMMAT reads data and write results on disk, whereas milorGWAS uses the RAM.

The Offset method is in *O*(*n*) while the AMLE and GMMAT are in $O(n^2)$, which makes this method appealing in terms of running times. However, in practice the initial step in $O(n^3)$ is the limiting factor: it would take more than 24 hours to fit the MLR model for $n = 50{,}000$. To manipulate larger samples such as those of biobanks, more complex workarounds and other approximate methods are needed [15].

### Type I error in the presence of population structure

We analyzed two data sets with a simulated binary phenotype. Simulations were based either on real genotype data from a recent GWAS [16], or on large cohort simulated with a coalescent model. Both data sets present cryptic relatedness and population stratification, with two strata (or two cohorts) with different disease prevalence. The simulations are fully described in the Methods section.

The first simulated set uses genotypes of 800 individuals from South Benin, ascertained in two sites distant of 20 km from each other, forming the two population strata in which we set different prevalences for simulating the phenotypes. These data were analyzed using the logistic regression (LR), the mixed logistic regression (MLR) (with Chen’s score test GMMAT or equivalently Wald test with AMLE) and the mixed linear model (MLM) (the status being analyzed as a quantitative trait with values 0 or 1), with or without the top 10 principal components (PCs) in the model.

**Fig. 2:**
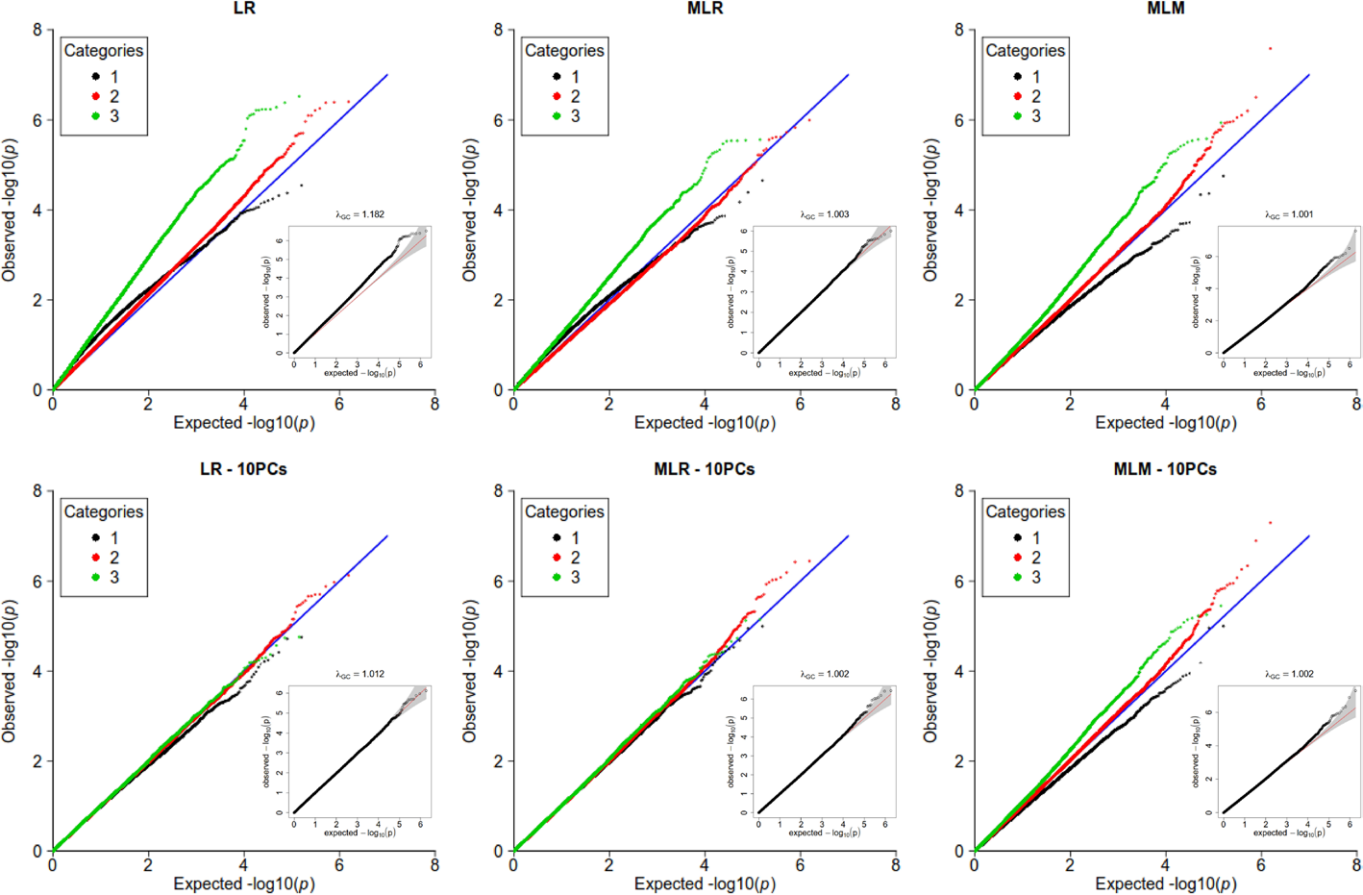
Simulated data based on South Benin genotypes (1). Stratified QQ-plots for logistic regression (LR), mixed logistic regression (MLR) using Chen’s score test (or AMLE), and mixed linear model (MLM) on the data simulated based on South Benin data. In each panel, a non-stratified QQ-plot is embedded. On the second row, 10 PCs were included as covariates. SNP categories are determined as in [14], based on the allele frequencies in the strata

The Fig. 2 displays the stratified QQ-plots of the corresponding *p* values, the 1, 847, 505 SNPs being split in three categories according to their allelic frequencies as described in the methods (we used a threshold $th = 0.8$). Category 1 contained $11.4\%$ of the SNPs, categories 2 and 3, respectively $77.6\%$ and $11.0\%$ of the SNPs.

When no PCs are included (first row of the Fig 1), statistic inflation is observed for LR ($\lambda = 1.182$). Based on the non-stratified QQ-plot, both MLM and MLR appear to adequately correct for population structure; however the stratified QQ-plot shows that this is not the case for SNPs in categories 1 and 3. In particular, for MLM, there is a statistic inflation for SNPs in category 3, and a deflation for SNPs in category 1.

When 10 PCs are included in the models (second row of the figure), this difference of behavior between SNPs categories persists for MLM. However, both logistic regression and MLR show an adequate correction for all categories of SNPs.

The second simulation set consists in 10, 000 individuals simulated on a $20\times 20$ grid, whose genotypes were obtained from a coalescent model. These data include some first order relatives. Ten millions independent SNPs were simulated, among which 2,840,903 had a minor allele frequency above $5\%$. Two strata were defined according to the individuals position on the grid (a “high risk strata” was defined as being the top left quarter of the grid), and a binary phenotype was simulated with different prevalences on these strata. Similar analyzes were performed on these data (Additional file 1: Figure 1). The 2,840,903 independent SNPs are at $23,7\%$ in category 1, $58.8\%$ in category 2 and $17.5\%$ in category 3. The same patterns of inflation and deflation of the test statistic are retrieved for most analyzes; the only notable difference is that, in this case, the logistic regression with 10 PCs does not correct the statistics inflation completely.

**Fig. 3:**
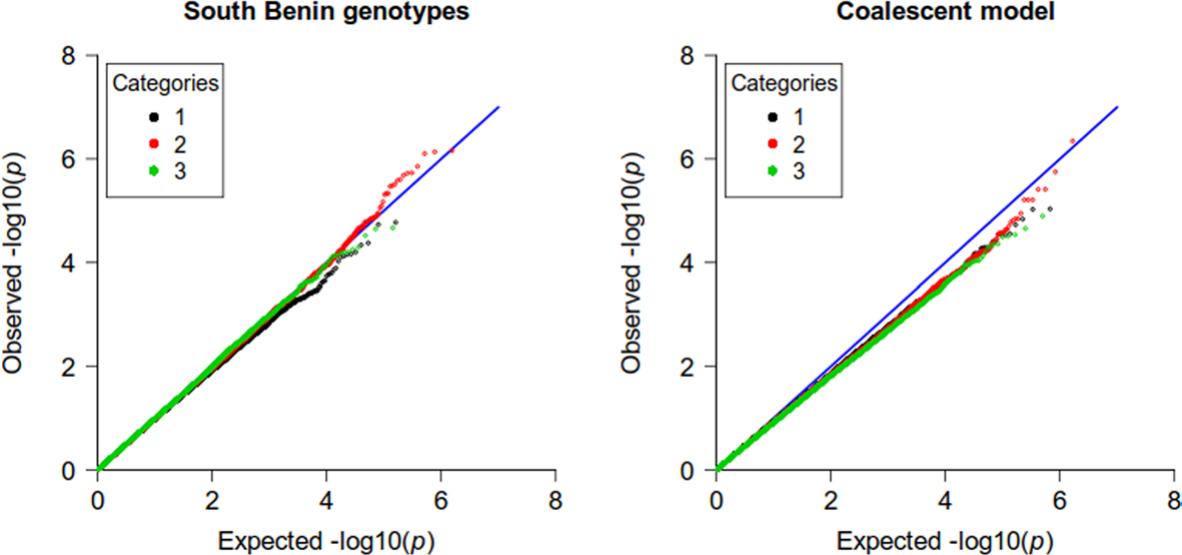
Simulated data based on South Benin genotypes (2). Stratified QQ-plots for the Offset method, for simulations based on South Benin data (left) and on the coalescent model (right). SNP categories are determined as in [14], based on the allele frequencies in the strata

Both data sets were also analyzed using the Offset method for MLR. Figure 3 shows the QQ-plots for the Offset method with the top 10 PCs included in the model, on the two simulations sets. While it adequately corrects for population structure in the data from the South Benin, it is too conservative in the case of the simulated cohort.

### Extension of the stratified QQ-plot

We extended the stratified QQ-plot proposed in [14] to the case in which strata are not clearly defined, or the strata information is missing. Our extension relies on the use of the first PCs instead of the strata.

**Fig. 4:**
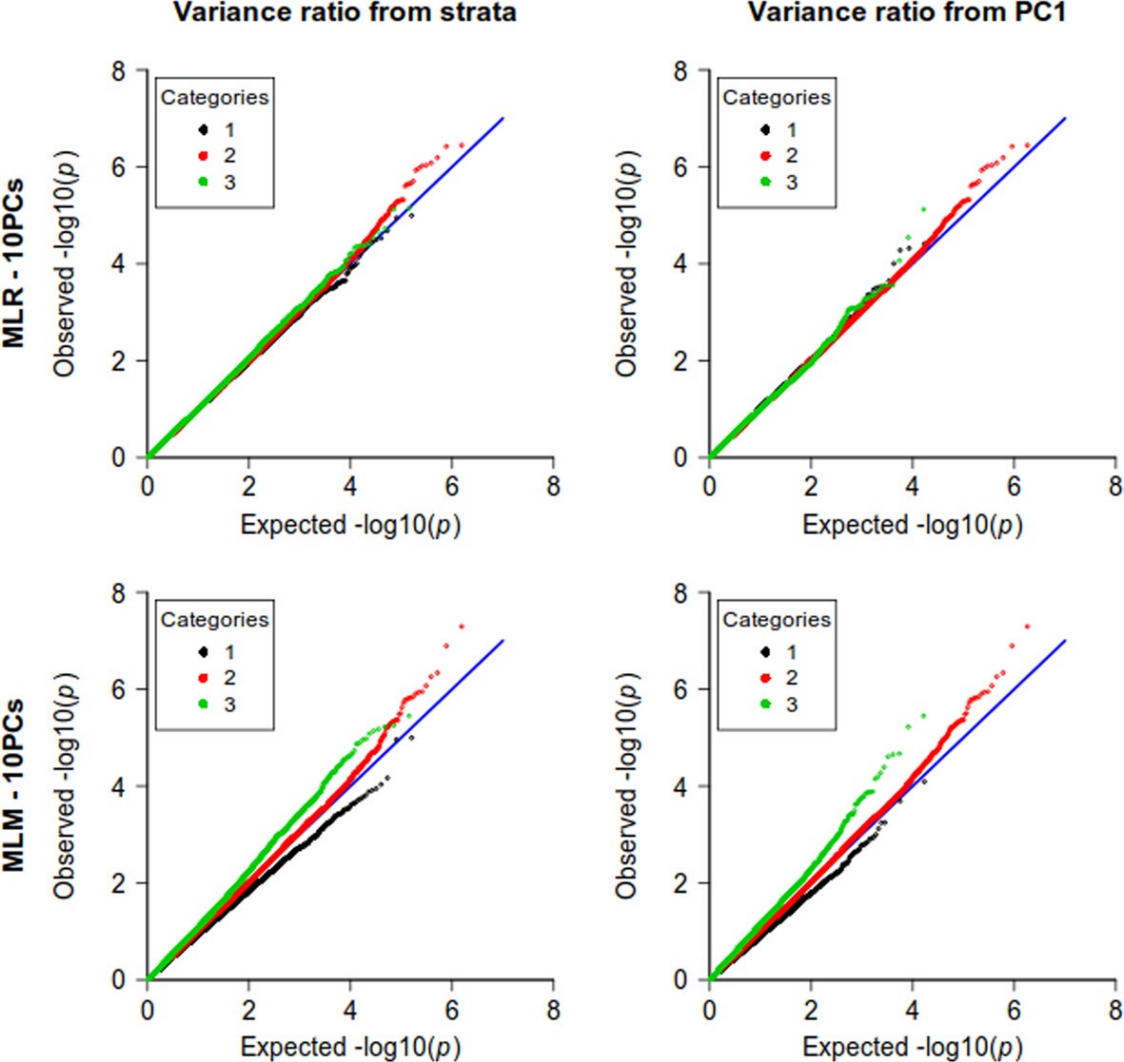
Stratified quantile–quantile plots based on PCs. Stratified QQ-plots obtained from the allele frequencies in the two strata (left) and from the first PC coordinates (right) for simulations based on South Benin data

**Fig. 5:**
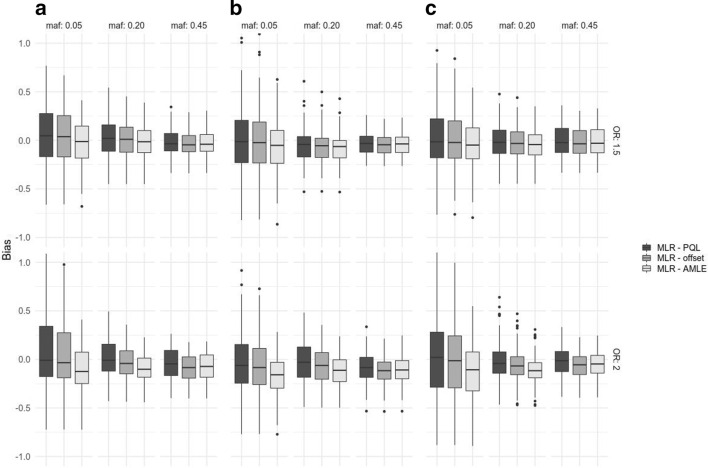
Bias of the SNP effect estimates ${\widehat{\gamma }}$ for two values of $\gamma$ [$\gamma = \log (1.5)$ and $\gamma = \log (2)$], in three scenarios (**a** moderate cohort and random effect; **b** moderate cohort effect, large random effect; **c** large cohort effect, moderate random effect), and three MAF bins (0.05 to 0.10, 0.20 to 0.25, and 0.45 to 0.50)

Figure 4 compares the stratified QQ-plots obtained using either strata information (as in Figs. 2 and 3) or the first PC, for two of the analyses already considered in Fig. 2, the MLM and the MLR, both including 10 PCs as fixed effects. The same comparisons were performed for analyses on the simulated cohort (Additional file 1: Figure 2). While there are small differences between the QQ-plots, we see that they allow similar diagnostics, that is, an incomplete correction of population structure for MLM analyses, and in contrast an adequate correction for MLR.

### Estimation of the SNPs’ effects

Figure 5 shows the bias $(\widehat{\gamma }- \gamma )$ obtained for two different values of $\gamma$, in three scenarios corresponding to different magnitude of cohort and random effects (A: moderate cohort and random effect; B: moderate cohort effect, large random effect; C: large cohort effect, moderate random effect). Three MAF bins were considered (from 0.05 to 0.10, from 0.20 to 0.25 and from 0.45 to 0.50).

In all situations, the PQL displays no bias, or a very small bias (for example in scenario B, corresponding to large random effects). The two proposed methods tend to have a negative bias. For data simulated with a moderate SNP effect $\gamma = 0.4$ (corresponding to $OR = 1.5$), they both have small negative biases (at most $-\,0.08$ corresponding to estimated $OR = 1.4$), independently of the population structure’s effect size (scenarios A, B and C).

For larger SNPs effects ($\gamma = 0.7$, corresponding to $OR = 2$), the bias increases, with AMLE having the larger bias, attaining in some situations $-\,0.1$ (corresponding to an estimated $OR = 1.8$). The bias is slightly more important for large random effects (scenario B).

**Table 1:** Comparison of powers in simulations based on South Benin data (800 individuals), with $\tau = 1$. $p_0$ and $p_1$ are the prevalences in the two strata

Scenario	$p_0$	$p_1$	OR	MAF bin	LR	AMLE	Offset
Moderate cohort effect	0.10	0.20	3	(0.20; 0.25]	0.179	0.261	0.268
Moderate cohort effect	0.10	0.20	3	(0.45; 0.50]	0.899	0.920	0.906
No cohort effect	0.30	0.30	2.5	(0.20; 0.25]	0.473	0.496	0.458
No cohort effect	0.30	0.30	2.5	(0.45; 0.50]	0.926	0.928	0.913

Powers at the genome-wide significance level $5\times 10^{-8}$ are reported for logistic regression (LR), AMLE (equivalent to the score test) and Offset methods, all analyses including 10 PCs with fixed effect

**Table 2:** Comparison of powers in simulations based on the coalescent model (5000 individuals), with $\tau = 1$

Scenario	$p_0$	$p_1$	OR	MAF bin	LR	AMLE	Offset
Large cohort effect	0.05	0.30	1.5	(0.20; 0.25]	0.307	0.251	0.202
Large cohort effect	0.05	0.30	1.5	(0.45; 0.50]	0.597	0.544	0.464
No cohort effect	0.30	0.30	1.5	(0.20; 0.25]	0.871	0.859	0.834
No cohort effect	0.30	0.30	1.5	(0.45; 0.50]	0.996	0.989	0.987

$p_0$ and $p_1$ are the prevalences in the two strata. Powers at the genome-wide significance level $5\times 10^{-8}$ are reported for logistic regression (LR), AMLE (equivalent to the score test) and Offset methods, all analyses including 10 PCs with fixed effect

### Power of the approximate methods

We compared the power of methods for which no inflation of type I error have been observed for all categories of SNPs. Table 1 reports powers obtained in simulations based on South Benin data for LR, AMLE (equivalent to the score test and GMMAT) and Offset methods. The AMLE performs slightly better than the two other methods, and in particular than LR.

Table 2 is dedicated to the simulations based on the coalescent model. Here LR has the largest power, which was expected as its type I error is largely inflated. Similarly, the Ofset method which was overconservative in this simulation setting, has a lowest power than AMLE.

### Illustration on real data

We tested the association with the presence of malaria infection during follow-up in the South Benin data. We focused on SNPs with a minor allele frequency greater than 0.05 on 100 kb segment on chromosome 20, and used both the AMLE and the PQL. The results are displayed in the Additional file 1: Figure 3 (Manhattan plot in panel a). The most associated SNPs (at $p = 10^{-4}$) is the same as in the GWAS (rs6124419). The association signal is weaker than in the original GWAS, which used the recurrence of infections in a Cox model.

The two methods give similar *p* values (panel b of the figure), but AMLE produces slightly higher *p* values for the most associated SNPs. Consistently, the largest $\beta$ values are slightly understimated by AMLE.

## Discussion

Our first result is a reproduction of the observation made by Chen et al. that is, in the presence of heterogeneity of disease prevalence between population strata, the mixed linear model (MLM) is inappropriate to analyse binary traits. MLM leads to conservative *p* values for some SNPs, and to anti-conservative *p* values for others, depending on the ratio of expected genotype variance in the two strata. The motivation of Chen et al. was a genome-wide association study of asthma including individuals from different Caribbean and Latin American backgrounds, with in particular *ca.* $15\%$ of individuals from Puerto Rico, in which the prevalence of asthma was much higher ($25.6\%$) than in other populations (from 3.9 to $9.6\%$). We retrieved similar results in an analysis of a simulated phenotype with large differences of prevalence among strata, based on genotype data from two geographically close cohorts (*ca.* 20 km apart) from South Benin [16], but with different self-reported ethnicities. Heterogeneity of prevalence may result from environmental factors (e.g. lifestyle, nutritional behavior, etc.), and could occur frequently in association studies, thus making the analysis of binary traits with the MLM incorrect, in particular in populations with a high genetic diversity, such as African populations.

A similar result was retrieved with data simulated with a coalescence model on a square grid, the “high-risk strata” consisting in the top left quarter of the grid; these simulations included many first-order related individuals and a random effect based on the kinship matrix. The mixed logistic regression (MLR) including the top PCs as fixed effects is the only method to completely correct for population structure in both simulations. A classical logistic regression including the top PCs can however be worth considering, as this conceptually simpler method was efficient enough for the South Benin data, in which the level of relatedness, though high, is lower than in the simulated data. In terms of power, however, our simulations hints towards a higher power of the MLR even in this case.

It is worth to note that in both simulations sets, it was necessary to include the top PCs as fixed effects in the MLR to obtain the correct type I error. The interest of including top PCs alongside the random components has been noted before [8, 10].

The diagnosis of the correctness of type I error cannot be based on the sole QQ-plot of *p* values, as the behavior of the test differs in SNP categories defined from the allelic frequencies in the two strata, as mentioned above. When these strata are clearly identified in the study, Chen et al. introduced a QQ-plot stratified on SNP categories based on the allele frequencies in the strata. We propose an extension of this method that can be used when population information is not available, using the first PC as a proxy (or any continuous variable defined at the population level, independently of the phenotype). Our simulations show that this method produces QQ-plot similar to those obtained with full knowledge of the two strata, thus allowing to diagnose better whether the population structure is adequately taken into account or not.

However, while the presence on the (stratified) QQ-plot of a deviation of the *p* values from their expected distribution implies that the association analysis is incorrect, the reverse is not necessarily true. Chen et al. demonstrated that new diagnostic plots can unveil hidden structures in the *p* values distribution; other new diagnostic plots could unveil other structures. More generally, while the correlation between polygenic effects is well modeled by the GRM, there is no reason that all unaccounted environmental variables have a correlation matrix similar to the GRM, and in theory the mixed model may prove inappropriate in some real studies. However, it seems to us that environmental variables that are not correlated to the genetic background would likely not be confounding variables.

Regarding SNPs’s effect estimation, made possible by the two proposed methods, our simulations studies show that both methods are slightly biased downward. The bias of the Offset method is less important than the bias of the AMLE, while the PQL has virtually no bias. It is known that in the presence of unaccounted heterogeneity, logistic regression effect estimates have negative biases [18–20]; our result hints that the heterogeneity between population strata is not fully taken into account by these methods. However, the bias is sensible only for large effects such as $OR = 2$, which makes its impact virtually negligible in GWAS. Note also that these methods are not adapted to the analysis of rare variants in highly unbalanced case-control ratio, as demonstrated in [15] for GMMAT – and thus for AMLE.

## Conclusion

We proposed two fast methods to estimate SNPs’ effects in mixed logistic regression. Both methods scale to up to at least 10,000 individuals, making them suitable for analysis of most GWAS data. Their implementation in an R package allows flexible use, with for example the possibility to specify a user defined GRM matrix.

The methods are constructed with conceptually simple mathematical principles which could be applied to other models (e.g. mixed Cox model in survival analysis), although literal computations to derive the formulas in the AMLE can be tedious. The Wald test performed with the AMLE is equivalent to the score test of [14], and thus the conclusions drawn for the MLR apply regarding type I error. The second method, which we called the Offset method, have similar performances on the simulations based on the South Benin genotypes, but is slightly over-conservative in the presence of strong familial effects in the simulations based on the coalescent model.

All methods are available in an R package **milorGWAS** based on the R package **Gaston** [21] for data manipulation. The R and C++ source code of **milorGWAS** is available on the CRAN at https://CRAN.R-project.org/package=milorGWAS. An excerpt of the data simulated using the coalescent model is also available, and is used in the package’s vignette to illustrate the methods.

## Methods

### Fast methods for mixed logistic regression

The mixed logistic regression model (or logistic mixed model) considered is

1 $$\begin{aligned} \mathop {\mathrm {logit}}E(Y) = \mathop {\mathrm {logit}}P(Y=1) = X\beta + G\gamma + \omega \end{aligned}$$

where *Y* is an *n*-dimensional vector of zeroes and ones ($\mathop {\mathrm {logit}}E(Y)$ is the vector of components $\mathop {\mathrm {logit}}E(Y_i)$), *X* is a $n \times p$ matrix of covariates (including a column of ones for the intercept), *G* is a vector of genotypes (ususally coded 0, 1 or 2), and $\omega$ is a random vector following a multivariate normal distribution $MVN(0, \tau K)$.

The likelihood of this model involves an integral over the random vector $\omega$. There is no closed-form for this integral, and numerical integration schemes are computationally intensive in high dimension. A classical approximate solution is the Penalized Quasi-Likelihood (PQL) algorithm, which is a sequence of approximations of the MLR model by linear mixed models. Even the PQL is too computationally intensive for estimating the effect $\gamma$ of all SNPs in a GWAS.

We describe below two approximate methods for estimating $\gamma$. The first is based on an approximation of the maximum likelihood estimate in the PQL. The second consists in first estimating predicted linear scores $X{\widehat{\beta }} + {\widehat{\omega }}$ in the MLR model (1) without the term $G\gamma$, and then incorporating these linear scores as an offset in a (non-mixed) logistic regression model.

#### Approximate Maximum Likelihood Estimate (AMLE)

We outline here the general principle on which the formula presented in Additional file 1 can be derived. This principle could be applied to any statistical model. Let $\ell (\kappa , \gamma )$ be a log-likelihood, in which $\kappa$ is a nuisance parameter and $\gamma$ is the parameter of interest. The null hypothesis to be tested is $H_0$ : $\gamma = 0$. Denote ${\widehat{\kappa }}_0$ the Maximum Likelihood Estimator (MLE) of $\kappa$ under the null hypothesis:

$$\begin{aligned} {\widehat{\kappa }}_0 = \mathop {\mathrm {arg}} \max _\kappa \ell (\kappa , 0). \end{aligned}$$

The score statistic to test for $H_0$ is the first derivative in $\gamma$ at the point $({\widehat{\kappa }}_0, 0)$ :

$$\begin{aligned} U(0) = {\partial \over \partial \gamma } \ell ({\widehat{\kappa }}_0, 0). \end{aligned}$$

The null hypothesis can be tested using $T = U(0)^2 / \mathop {\mathrm {var}}(U(0))$, which asymptotically follows a $\chi ^2(1)$ distribution. A second-order approximation, with $\kappa = {\widehat{\kappa }}_0$ fixed, gives

$$\begin{aligned} \ell ({\widehat{\kappa }}_0, \gamma ) \simeq \ell ({\widehat{\kappa }}_0, 0) + {\partial \over \partial \gamma } \ell ({\widehat{\kappa }}_0, 0) \gamma + {1\over 2} \cdot {\partial ^2 \over \partial \gamma ^2} \ell ({\widehat{\kappa }}_0, 0) \gamma ^2 \end{aligned}$$

which maximizes in

$$\begin{aligned} {\widehat{\gamma }} = -{{\partial \over \partial \gamma } \ell ({\widehat{\kappa }}_0, 0) \over {\partial ^2 \over \partial \gamma ^2} \ell ({\widehat{\kappa }}_0, 0) } . \end{aligned}$$

This estimator of ${\widehat{\gamma }}$ is not the MLE of $\gamma$, but when the true value of $\gamma$ is small enough, both estimators are close.

In the context of GWAS, $\kappa$ is the vector of random term variance and covariates effects, while $\gamma$ is the effect of the SNP to be tested. This estimator shares with the score test the advantage that ${\widehat{\kappa }}_0$ has to be estimated only once; the partial derivatives in $\gamma$ are usually easy to compute, allowing a fast testing and estimating procedure.

However, as mentioned above, in the case of the MLR, the likelihood can’t be computed efficiently. We use the PQL to estimate the nuisance parameter $\kappa = (\beta , \tau )$, and the log-likelihood of the last linear approximation used in the PQL to estimate $\gamma$. The variance of the resulting ${\widehat{\gamma }}$ is estimated in this linear approximation; the resulting Wald test is identical to the score test of [14]. All details are given in the Additional file 1.

#### Offset

The proposed method consists of estimating a vector of individual effects, including both the random components and the covariates in *X*, which is then incorporated in a logistic regression as an offset:

- First estimate ${\widehat{\beta }}_0$ and ${\widehat{\omega }}_0$ under the hypothesis $\gamma = 0$, in the MLR model
  $$\begin{aligned} \mathop {\mathrm {logit}}E(Y) = X\beta + \omega \end{aligned}$$
  with $\omega$ as in (1).
- Then, for each vector of genotypes *G*, fit a linear model for
  $$\begin{aligned} E(G) = X\delta , \end{aligned}$$
  let ${{\widetilde{G}}} = G - X{\widehat{\delta }}$ be the residuals of *G*, and estimate $\gamma$ in the fixed-effects logistic regression
  $$\begin{aligned} \mathop {\mathrm {logit}}E(Y) = X{\widehat{\beta }}_0 + {\widehat{\omega }}_0 + G \gamma , \end{aligned}$$
  in which the vector $X{\widehat{\beta }}_0 + {\widehat{\omega }}_0$ is an offset (that is, is held constant).

The motivation of this heuristic is that a similar two-steps method applied to a linear model $E(Y) = X\beta + G\gamma$ would give the same estimator ${\widehat{\gamma }}$ than the classical regression (cf Additional file 1 for the details).

#### Asymptotic complexity of the methods and efficiency of the implementation

The initial step of both AMLE and Offset method is to fit the MLR model $\mathop {\mathrm {logit}}E(Y) = X\beta + \omega$. Each iterative step of the PQL needs the inversion of an $n \times n$ matrix, where *n* is the number of individuals; the complexity of this operation is $O(n^3)$. The second step of the AMLE involves, for each SNP, multiplying an $n \times n$ matrix *P* by the vector of genotypes; this is the most costly operation, and the complexity of this step is thus $O(n^2)$. The second step of the Offset method is an iterative algorithm, each iteration of which is in *O*(*n*) (considering the number of covariates as fixed). The complexity of the second step of the Offset is thus *O*(*n*).

Our package milorGWAS is implemented in C++ using Rcpp [22], and RcppEigen [23] for matrix arithmetic. We performed simulations with random genotypes for sample size $n = 1000$, $n = 2000$ and $n = 5000$, to assess the performance of the implementation.

### Stratified QQ-plot

One of the contributions of Chen et al. was to show that a QQ-plot of log *p* values was not sufficient to diagnose an incorrect test procedure, and to propose a “stratified QQ-plot” in which different categories of SNPs are represented separately. This allowed to see that in some of these categories, the test statistics are either inflated or deflated, while the overall distribution of *p* values was correct. Here is how their categories were defined. Chen et al. consider a population with two strata, indexed by $i = 0$ or 1 according to disease prevalence in ancestry groups, $i = 1$ being the group with a higher risk of desease. The strata are assumed to be panmictic, so that expected variance of a SNP genotype *G* in stratum *i* is $\mathop {\mathrm {var}}_i (G) = 2 p_i q_i$ , $p_i$ and $q_i$ being the SNP allele frequencies. Each SNP is categorized according to the variance ratio $r(G) = \mathop {\mathrm {var}}_1(G) / \mathop {\mathrm {var}}_0(G)$ between the two strata as follows (Chen et al. use a threshold $\mathop {th} = 0.8$):

- The SNPs with $r(G) < \mathop {th}$ are category 1,
- the SNPs with $\mathop {th} \le r(G) \le 1/\mathop {th}$ are category 2,
- the SNPs with $1/\mathop {th} < r(G)$ are category 3.

We propose to extend the method to stratify QQ-plots according to any covariate *Z*. If $G \in \{0, 1, 2\}^n$ is the vector of genotypes, *Z* a vector with components in the range [0, 1], and ${\mathbf {1}}$ denotes a vector of ones, we let

$$\begin{aligned} q_1 = {1\over 2} {Z'G \over Z' {\mathbf {1}}} \text { and } q_0 = {1\over 2} {({\mathbf {1}} - Z)' G \over ({\mathbf {1}} - Z)' {\mathbf {1}}}, \end{aligned}$$

and we defined SNP categories as above (with $p_i = 1 - q_i$). If *Z* is the indicator variable of the strata, $q_1$ and $q_0$ are the allelic frequencies in the two strata, and the categories will be identical to those of Chen et al. The point of this extension is that when the relevant sub-strata are unknown, one could use one of the top genomic PCs instead (after rescaling them to [0, 1]).

### Simulation studies: type I error in the presence of population structure

We performed two sets of simulations, based on the simulations performed in [14], to assess the efficiency of the different methods to correct for population stratification. In both simulation sets, there are two strata (or two cohorts) with different disease prevalence, and related individuals. Simulations were performed under the null hypothesis of no genetic association [$\gamma = 0$ in Eq. (1)] and were analyzed with

- a logistic regression model (LR)
- a mixed linear model (MLM)
- a mixed logistic regression model, using Chen et al. score test GMMAT, identical to AMLE Wald test (MLR)
- a mixed logistic regression model, using the offset method (Offset)

All analyses were repeated with the top ten PCs included as fixed effects in the model. We assessed the capacity of each test procedure to control type I error rates using Chen’s stratified QQ-plot. Moreover, to gauge the interest of our extension, we compared Chen’s QQ-plot to the stratified QQ-plot obtained using the first PC instead of the cohort indicator.

#### Simulations based on South Benin data

We used genotype data from a GWAS on mild malaria susceptibility performed on two cohorts in South Benin [16]. The participants were ascertained in two sites distant of 20 km from each other, in three different health centers for the first cohort and two for the second one. After quality control (QC), the genotypes of 800 individuals were available, 525 in the first cohort, and 275 in the second one. The genotyping was performed with Illumina HumanOmni5 chips (1,847,505 SNPs after QC and filtering out SNPs with minor allele frequency, MAF, less than $5\%$). This genetic sample presents both population structure and cryptic relatedness. Self-reported ethnic composition differed between the two cohorts, and principal component analysis confirmed the presence of population structure. A sub-structure related to the health center, where the participant was ascertained, was also apparent. Moreover, substantial relatedness was observed in the sample, with levels of relationship corresponding to half-sibs, uncle-nephew or even 3/4 siblings for some pairs (estimated kinship coefficient $\phi$ from 0.10 to 0.16).

We simulated a binary phenotype with a difference of disease prevalence between the two cohorts, and a random effect modeling both population stratification and relatedness. Specifically, the probability on an individual *i* of being a case was calculated as:

2 $$\begin{aligned} \mathop {\mathrm {logit}}(p_i) = a_0 + a_1 Z_i + \omega _i \end{aligned}$$

where *Z* is an indicator variable for belonging to the second cohort, and $\omega _i$ an individual random effect. The coefficients $a_0$ and $a_1$ were defined as $a_0 = \mathop {\mathrm {logit}}(0.05)$ and $a_1 = \mathop {\mathrm {logit}}(0.30) - \mathop {\mathrm {logit}}(0.05)$, so as to obtain a prevalence of 0.05 in the first cohort and of 0.30 in the second one (not taking into account the presence of random effects). The vector of random effects was simulated following a multivariate normal distribution:

$$\begin{aligned} \omega \sim {\mathcal {N}} (0, \tau K ) \end{aligned}$$

where *K* is the Genomic Relationship Matrix (GRM) calculated from all the SNPs. We set $\tau = 1$.

#### Simulations based on a coalescent model

We also performed coalescent simulations, reproducing closely the simulations described in [14], to obtain genotypes for a large cohort of 10,000 individuals, with both population structure and relatedness, using the ms software [17]. This procedure, which is based on a stepping stone model with symmetric migration between adjacent cells of the grid, is commonly used to simulate a population with a spatially continuous population structure [24, 25]. We use a $20 \times 20$ grid, in which the migration rate between adjacent cells was set to 10; this parameter produces a Wright’s fixation index $F_{st} < 0.01$ when dividing the simulated grid into two equal sub-populations, a level comparable to what is observed within Europe [24]. We simulated a total of 10 million independent SNPs. After filtering out SNPs with MAF lower than $5\%$, 2,840,903 SNPs were available. The full command line arguments for ms are included in Additional file 1. We also created a R data package containing part of the simulated data (link in Additional file 1).

To obtain related individuals, we first simulated the genotypes for 8000 founders (20 on each of the 400 cells). We then sampled 10 pairs of individuals in each cell, forming 4000 couples, and simulated two offsprings by gene dropping. Thus we obtained 16,000 individuals (founders and offspring) from which 10,000 individuals were randomly selected to obtain the cohort.

The phenotype was simulated as before using Eq. (2), where $Z_i$ was set to one when individuals were sampled in the top left $10 \times 10$ grid, corresponding to strata with a higher risk. The values of $a_0$, $a_1$ and $\tau$ were set as before; in this simulation set, $K = 2\Phi$ were $\Phi$ is the matrix of kinship coefficients (entries are 0.5 for first order relatives, 1 on the diagonal, 0 elsewhere). Data analyses were subsequently performed using a GRM calculated from 100,000 random SNPs.

### Simulations studies: estimation of the SNPs’ effects

Simulations including a SNP effect were performed using the South Benin data set, using the model

$$\begin{aligned} \mathop {\mathrm {logit}}(p) = a_0 + a_1 Z + G \gamma + \omega \end{aligned}$$

with *G*, a genotype picked at random in the data, and *Z* and $\omega$ as described above. We considered four different scenarios:

A Moderate cohort effect (respective prevalence $p_0 = 0.10$ and $p_1 = 0.20$) and moderate random effect ($\tau = 0.3$).
B Moderate cohort effect (respective prevalence $p_0 = 0.10$ and $p_1 = 0.20$) and large random effect ($\tau = 1$).
C Large cohort effect (respective prevalence $p_0 = 0.05$ and $p_1 = 0.30$) and moderate random effect ($\tau = 0.3$).

The coefficients $a_0$ and $a_1$ are computed as $a_0 = \mathop {\mathrm {logit}}(p_0)$ and $a_1 = \mathop {\mathrm {logit}}(p_1) - \mathop {\mathrm {logit}}(p_0)$; *G* is centered to ensure that the expected prevalence is as prescribed. For each scenario, we considered SNPs with MAF in intervals (0.05; 0.10], (0.20; 0.25] and (0.45; 0.50], and SNP effect $\gamma = \log (1.5)$ and $\gamma = \log (2)$ (corresponding to $OR = 1.5$ and 2). One hundred replicates were performed for each condition, redrawing a vector of random effects each time, and analyzed with the PQL, the Offset and the AMLE, including the top 10 PCs.

### Simulations studies: comparison of powers

To compare the power of the methods that have shown a correct type I error, we performed additional simulations in a similar setting as in the previous section, with a large random effect ($\tau = 1$), a moderate cohort effect (as defined above) and $OR = 3$, and without cohort effect and $OR = 2.5$. Other simulations were performed based on 5000 individuals extracted from the cohort generated under the coalescent model, with either a large cohort effect or no cohort effect, and an $OR = 1.5$. In each scenario, 1000 replicates were performed, redrawing a vector of random effects each time.

### Illustration on real data

To illustrate the method, we applied it on the data from the GWAS in South Benin described in [16]. We used as binary phenotype the presence/absence of any malaria infection during the follow-up (there were 229 individuals with no infection and 546 with at least one infection). We tested all SNPs with a minor allele frequency greater than 0.05 on a 100 kb segment on chromosome 20, which contains one of the strongest association signals discovered in [16]. We included as covariates the site of ascertainment, the duration of the wollow-up, and mean infection exposure. We performed the testing with AMLE and, for comparison, with the PQL.

## Supplementary information

**Additional file 1.** Including details on the AMLE and Offset methods, commands used for the simulations with the coalescent model and supplementary figures.

## Data Availability

The genotype datasets from South Benin used in the current study are available in the DataSuds repository (10.23708/EXSQTM). The dataset are not publicly available due to patient confidentiality but are available for researchers who meet the criteria for access to confidential data. Data to reproduce the simulations based on a coalescent model are included in this published article and its supplementary information files.
