# Supplementary material for: Mixed logistic regression in genome-wide association studies
Source: BMC Bioinformatics. 2020 Nov 23;21:536. doi: 10.1186/s12859-020-03862-2 (PMC7684894; doi:10.1186/s12859-020-03862-2)
Supplement: Supplementary file 1 — Additional file 1. Including details on the AMLE and Offset methods, commands used for the simulations with the coalescent model and supplementary figures. [file 12859_2020_3862_MOESM1_ESM.pdf]

Mixed Logistic Regression in Genome-Wide Association Studies

Supplementary materials

Jacqueline Milet and Hervé Perdry

There are three sections in these supplementary materials:

A. Details on the AMLE and Offset methods

B. Commands used for the simulations with the coalescent model

C. Supplementary figures

A. Details on AMLE and Offset methods

In section 1, we give some background on the PQL algorithm, which is needed to understand the construction of the AMLE formula. In section 2, we give a detailed derivation of the AMLE formula. In section 3, we justify briefly the Offset method by a heuristic argument.

For the sake of simplicity, if $u, v \in \mathbb{R}^n$ we denote by $f(u)$ the vector of $\mathbb{R}^n$ with components $f(u_i)$, by $v(1-v)$ the vector with components $v_i(1-v_i)$, by $\frac{u}{v}$ the vector with components $\frac{u_i}{v_i}$.

1 The PQL algorithm

1.1 Background: the Restricted Likelihood for linear mixed model

Consider a linear mixed model with parameters β and θ

$$Y = X\beta + \varepsilon$$

were $Y \in \mathbb{R}^n$, $X \in \mathbb{R}^{n\times p}$, and ε is multivariate normal with null expected value and variance $\mathrm{var}(\varepsilon) = V(\theta)$ depends linearly on θ. A classical case is $\varepsilon = \omega + e$ with $\omega \sim MVN(\tau K)$ and $e \sim MVN(\sigma^2 I_n)$; in that case, the variance parameter is $\theta = (\tau, \sigma^2)$, and $V(\theta) = \tau K + \sigma^2 I_n$.

1.1.1 Restricted Likelihood

The privilegied approach to estimate parameters of this model is to maximize the restricted likelihood (RL). The RL is obtained by projecting the model on a subspace orthogonal to all the covariates in X. More precisely, let C be a contrast matrix for X, that is a $(n-p)\times n$ matrix with $CX = 0$ and $CC' = I_{n-p}$. Consider the restricted model

$$CY = CX\beta + C\varepsilon = C\varepsilon,$$

with $\mathrm{var}(C\varepsilon) = CVC'$, which still depends linearly on θ.

The log-likelihood of this model is the Restricted Likelihood

$$RL(\theta) = -\frac{1}{2}\log|CVC'| - \frac{1}{2}Y'PY$$

where $P = C\left(CVC'\right)^{-1}C'$. It can be verified (Searle et al, 2009) that $\log|CVC'|$ and P do not depends on the choice of the matrix C: $\log|CVC'| = \log|V| + \log\left|X'V^{-1}X\right| + \text{constant}$ and

$$P = V^{-1} - V^{-1}X\left(X'V^{-1}X\right)^{-1}X'V^{-1}.$$

Finally

$$RL(\theta) = -\frac{1}{2}\log|V| - \frac{1}{2}\log\left|X'V^{-1}X\right| - \frac{1}{2}Y'PY$$

can be maximized using an iterative algorithm (a classical choice is AIREML, for Average Information Restricted Maximum Likelihood).

1.1.2 Estimators and predictors

Once the estimate of $\widehat{\theta}$ has been obtained, estimates of β are obtained as $\widehat{\beta} = \left(X'V^{-1}X\right)^{-1}X'V^{-1}Y$. Predictors of the random terms can be also computed. For example, in the aforementioned case where $\varepsilon = \omega + e$ with $\text{var}(\omega) = \tau K$ and $\text{var}(e) = \sigma^2 I_n$, we have $\widehat{\omega} = \widehat{\tau}K\widehat{P}Y$ and $\widehat{e} = \widehat{\sigma^2}\widehat{P}Y$, with $\widehat{P} = P\left(\widehat{\theta}\right)$. The equality $Y = X\widehat{\beta} + \widehat{\omega} + \widehat{e}$ holds. These predictors are known as the Best Linear Unbiased Predictors (BLUP) of the random terms.

1.2 Logistic mixed model: the PQL algorithm

The PQL algorithm is a generalization of the Iteratively Reweighted Least Squares algorithm, which can be used to fit logistic regressions, to the mixed logistic model. It can be further justified with a Laplace approximation argument (Breslow et al, 1993). We show here how it can be obtained simply as successive linear approximations of the mixed effects logistic model.

We denote classically by logit the function $\text{logit}(p) = \log(p) - \log(1-p)$, and its reciprocal by $\text{expit}(x) = (1 + e^{-x})^{-1}$. We have observations $Y_i \in \{0, 1\}$ indexed by $i = 1, \ldots, n$, with a vector $X_i \in \mathbb{R}^{1\times p}$ of predictors. Consider the model

$$\text{logit}\, E(Y_i) = X_i\beta + \omega_i \quad (i = 1, \ldots, n)$$

where $\beta \in \mathbb{R}^p$ are the so-called fixed effects, and the ω_i are the random effects ; the vector of all random effects is taken in a multivariate normal distribution $\omega \sim MVN(0, \tau K)$, with $K \in \mathbb{R}^{n\times n}$.

Denote $\pi_i = \text{expit}(X_i\beta + \omega_i)$. We have $E(Y_i|\omega) = \pi_i$ and $\text{var}(Y_i|\omega) = (\pi_i(1-\pi_i))$.

Given provisional estimates $\widehat{\beta}$ and $\widehat{\omega}$ of β and ω, let $\widehat{\pi}_i = \text{expit}\left(X_i\widehat{\beta} + \widehat{\omega}_i\right)$ and

$$Z_i = X_i\widehat{\beta} + \widehat{\omega}_i + \frac{Y_i - \widehat{\pi}_i}{\widehat{\pi}_i(1-\widehat{\pi}_i)}.$$

The vector $Z = (Z_1, \ldots, Z_n)$ is the *pseudo-response* vector.

Expected value of the pseudo response. We have

$$\begin{aligned} E(Z_i|\omega) &= X_i\widehat{\beta} + \widehat{\omega}_i + \frac{1}{\widehat{\pi}_i(1-\widehat{\pi}_i)}\left(E(Y_i|\omega) - \widehat{\pi}_i\right) \\ &= X_i\widehat{\beta} + \widehat{\omega}_i + \frac{1}{\widehat{\pi}_i(1-\widehat{\pi}_i)}\left(\pi_i - \widehat{\pi}_i\right). \end{aligned}$$

A first order approximation in the neighbourhood of $\widehat{\beta}$ and $\widehat{\omega}$ gives

$$\pi_i = \text{expit}(X_i\beta + \omega_i) \simeq \widehat{\pi}_i + \widehat{\pi}_i(1-\widehat{\pi}_i)\left(X_i\left(\beta - \widehat{\beta}\right) + (\omega_i - \widehat{\omega}_i)\right)$$

thus

$$E(Z_i|\omega) \simeq X_i\beta + \omega_i$$

and $E(Z_i) \simeq X_i\beta$, thus $E(Z) \simeq X\beta$.

Variance of the pseudo response. We have

$$\text{var}(Z_i|\omega) = \left(\frac{1}{\widehat{\pi}_i(1-\widehat{\pi}_i)}\right)^2 \pi_i(1-\pi_i),$$

and if π_i is close to $\widehat{\pi}$, $\text{var}(Z_i|\omega) \simeq \left(\frac{1}{\widehat{\pi}_i(1-\widehat{\pi}_i)}\right)$. Let W be the diagonal matrix $W = \text{diag}\left(1/(\pi_i(1-\pi_i))\right)$; we have $\text{var}(Z|\omega) \simeq W$ and finally

$$\text{var}(Z) = \text{var}(E(Y|\omega)) + E(\text{var}(Y|\omega)) \simeq \text{var}(\omega) + W = \tau K + W.$$

Iterations. The pseudo-response Z follows then approximately a linear mixed model

$$Z = X\beta + \varepsilon,$$

with $\text{var}(\varepsilon) = \tau K + W$. This model is used to obtained new estimates of β and ω, by the restricted maximum likelihood (REML) method, as described in section 1.1. The process is iterated until convergence.

Remark 1. Let Z be the pseudo-response computed at the last step, and $\widehat{P} = P(\widehat{\tau})$ be the matrix obtained in the corresponding REML optimization. The theory of BLUPs (sketched in section 1.1.2) implies that $Z = X\widehat{\beta} + \widehat{\omega} + W\widehat{P}Z$ (with $\widehat{\omega} = \widehat{\tau}K\widehat{P}Z$). However $Z = X\widehat{\beta} + \widehat{\omega} + W(Y - \widehat{\pi})$, and if the convergence occured, we have

$$W\widehat{P}Z = W(Y - \widehat{\pi}),$$

from which $\widehat{P}Z = Y - \widehat{\pi}$ follows readily.

Remark 2. In the absence of random effects this procedure is known as Iterated Reweighted Least Squares, and converges towards the maximum likelihood estimates of the logistic regression. In the mixed case, this is no longer true.

2 The AMLE

2.1 Estimator of the SNPs' effects

The model considered is now

$$\text{logit}\, E(Y) = X\beta + G\gamma + \omega$$

where Y, X, ω are as in section 1.2 (excepted that X now contains only covariates other than the SNP to test), and $G \in \mathbb{R}^n$ is the vector of genotypes.

The analysis consists in two steps. In the first step, the null model in which $\gamma = 0$ has been fitted by the PQL algorithm described above. Let Z be the pseudo-response computed at the last PQL iteration, and $\widehat{V}$ and $\widehat{P}$ the associated matrix. We have noted in section 1.2 that $\widehat{P}Z = Y - \widehat{\pi}$.

The second step, carried on for each SNP, estimates γ by

$$\widehat{\gamma} = (G'\widehat{P}G)^{-1}G'\widehat{P}Z = (G'\widehat{P}G)^{-1}G'(Y - \widehat{\pi}).$$

and tests the hypothesis $\gamma = 0$ based on $\text{var}(\widehat{\gamma}) = (G'\widehat{P}G)^{-1}$, which leads to the 1 degree of freedom chi-square statistic

$$\frac{(\widehat{\gamma})^2}{\text{var}(\widehat{\gamma})} = (G'\widehat{P}G)^{-1}\left(G'(Y - \widehat{\pi})\right)^2,$$

identical to the score test of Chen et al.

2.2 Justification of the formula

We give hereafter mathematical arguments in favor of this formula, which can be found without proof in Zhou et al. (2015). Let Z the pseudo response at convergence of the null model, and C a contrast matrix for X. The estimate of γ is obtained in the linear mixed model

$$Z = X\beta + G\gamma + \xi$$

where $\text{var}(Z) = \text{var}(\xi) = \widehat{\tau}K + W = V$; or more precisely in the restricted model

$$CZ = CX\beta + CG\gamma + C\xi = CG\gamma + C\xi.$$

We have $E(CZ) = CG\gamma$ and $\text{var}(CZ) = \text{var}(C\xi) = CVC'$.

Using the matrix $P = C\left(CVC'\right)^{-1}C'$ defined in section 1.1, the log-likelihood of this model can be written

$$\ell(\gamma, \tau) = -\frac{1}{2}\log|V| - \frac{1}{2}\log\left|X'V^{-1}X\right| - \frac{1}{2}(Z - G\gamma)'P(Z - G\gamma).$$

Its partial derivative in γ is

$$\frac{\partial \ell}{\partial \gamma} = G'P(Z - G\gamma)$$

and thus we get $\widehat{\gamma} = (G'PG)^{-1}G'PZ$. We use for P the matrix $\widehat{P}$ obtained at convergence of the null model; then

$$\widehat{\gamma} = (G'\widehat{P}G)^{-1}G'\widehat{P}Z = (G'\widehat{P}G)^{-1}G'(Y - \widehat{\pi}).$$

Its variance is $\text{var}(\widehat{\gamma}) = (G'PG)^{-1}G'P \cdot V \cdot PG(G'PG)^{-1} = (G'PG)^{-1}$ as $PVP = P$ (as can be seen readily from the definition of P).

3 The Offset method

The Offset method consists in two steps as well. The first step is identical to the first step of the AMLE: the model

$$\text{logit}\, E(Y) = X\beta + \omega$$

with $\text{var}(\omega) = \tau K$ is fitted using the PQL; denote by $\widehat{\beta}^{(0)}$ be the estimate of β and by $\widehat{\omega}^{(0)}$ the BLUP of ω.

The second step, for each SNP with genotype vector G, consists in

- computing $\tilde{G}$ the residual of the linear regression of G by X
- estimating γ by fitting the (non-mixed) logistic regression model

$$\text{logit}\, E(Y) = \left(X\widehat{\beta}^{(0)} + \widehat{\omega}^{(0)}\right) + \tilde{G}\gamma$$

  where $\left(X\widehat{\beta}^{(0)} + \widehat{\omega}^{(0)}\right)$ is an offset.

We justify this heuristic by showing that in the case of a linear regression, this approach produces the right estimate for γ.

3.1 Motivation: The linear case

In the linear case, the regression $E(Y) = X\beta + G\gamma$ where $X \in \mathbb{R}^{n\times p}$ and $G \in \mathbb{R}^{n\times 1}$ can be performed in two steps:

1. Let $\widehat{\beta}^{(0)}$ be the estimate of β in the restricted model
$$E(Y) = X\beta$$
2. Let $\tilde{G}$ be the residual of the regression of G by X. Estimate γ in the model
$$E(Y) = X\widehat{\beta}^{(0)} + \tilde{G}\gamma$$
where $\widehat{\beta}^{(0)}$ is held constant ($X\widehat{\beta}^{(0)}$ is an offset).

The estimate $\widehat{\gamma}$ of γ obtained in this way is identical to the estimate obtained through the classical one step procedure. Note that the presence of an offset in the last regression above is an other way to state that we're doing the regression of the residual $\tilde{Y} = Y - X\widehat{\beta}^{(0)}$ of Y by $\tilde{G}$:
$$E(\tilde{Y}) = \tilde{G}\gamma.$$

Proof. The estimated coefficients of regression of Y on X and G are caracterized by the fact that the residual $Y - (X\widehat{\beta} + G\widehat{\gamma})$ is orthogonal to X and G, that is
$$X'X\widehat{\beta} + X'G\widehat{\gamma} = X'Y$$
$$G'X\widehat{\beta} + G'G\widehat{\gamma} = G'Y.$$

Similarly, in the first step $\widehat{\beta}^{(0)}$ verifies
$$X'X\widehat{\beta}^{(0)} = X'Y. \tag{1}$$

In the second step, denoting by $\widehat{b}$ the coefficients of regression of G on X, we have
$$X'X\widehat{b} = X'G. \tag{2}$$

Then the final regression of $\left(Y - X\widehat{\beta}^{(0)}\right)$ by $\tilde{G} = \left(G - X\widehat{b}\right)$ is caracterized by
$$\left(G - X\widehat{b}\right)'\left(G - X\widehat{b}\right)\widehat{\gamma} = \left(G - X\widehat{b}\right)'\left(Y - X\widehat{\beta}^{(0)}\right),$$
that is
$$\left(G'G - 2\widehat{b}'X'G + \widehat{b}'X'X\widehat{b}\right)\cdot\widehat{\gamma} = G'Y - G'X\widehat{\beta}^{(0)} - \widehat{b}'X'Y + \widehat{b}'X'X\widehat{\beta}^{(0)}$$

By pluging (1) and (2) in this equation, we have
$$\left(G'G - \widehat{b}'X'G\right)\cdot\widehat{\gamma} = G'Y - G'X\widehat{\beta}^{(0)}. \tag{3}$$

Finally, we prove that the coefficients of regression of Y and G are $\widehat{\beta} = \widehat{\beta}^{(0)} - \widehat{b}\cdot\widehat{\gamma}$ and $\widehat{\gamma}$. We have
$$\begin{aligned} X'X\left(\widehat{\beta}^{(0)} - \widehat{b}\cdot\widehat{\gamma}\right) + X'G\widehat{\gamma} &= X'X\widehat{\beta}^{(0)} - \left(X'X\widehat{b} - X'G\right)\cdot\widehat{\gamma} \\ &= X'Y \end{aligned}$$
using (1) and (2), and
$$\begin{aligned} G'X\left(\widehat{\beta}^{(0)} - \widehat{b}\cdot\widehat{\gamma}\right) + G'G\widehat{\gamma} &= G'X\widehat{\beta}^{(0)} + \left(G'G - G'X\widehat{b}\right)\cdot\widehat{\gamma} \\ &= G'Y \end{aligned}$$
using (3).

B. Simulations with ms

Our simulations can be reproduced by creating a bash script `mkparams.sh` containing the following code:

```
#!/bin/bash
echo -n "-s 1 -I 400"
for i in {0..399}
do
  echo -n "40 "
done
echo -n "-ma "
for i in {0..399}
do
  x1=$((i/20 + 1))
  y1=$((i%20 + 1))
  for j in {0..399}
  do
    x2=$((j/20 + 1))
    y2=$((j%20 + 1))
    dd=$(( (x1-x2)*(x1-x2) + (y1-y2)*(y1-y2) ))
    if [ $i == $j ]
    then
      echo -n "x "
    elif [ $dd == 1 ]
    then
      echo -n "10 "
    else
      echo -n "0 "
    fi
  done
done
```

This script is used to generate the parameter files, and `ms` is run as follows:

```
./mkparams.sh > params.txt
ms 16000 1e7 -f params.txt
```

The program `ms` is described in Hudson (2002), Generating samples under a Wright–Fisher neutral model of genetic varia- tion, Bioinformatics 18(2), and was retrieved from `http://home.uchicago.edu/~rhudson1/source/mksamples.html`.

A R data package was created, containing a part of the data (1 000 individuals, 600 000 SNPs) for demonstration purposes. This package can be installed in R with

```
install.packages("GridData", repos="https://genostats.github.io/R/")
```

Its use is illustrated in the vignette of the package milorGWAS (on CRAN).

C. Supplementary figures

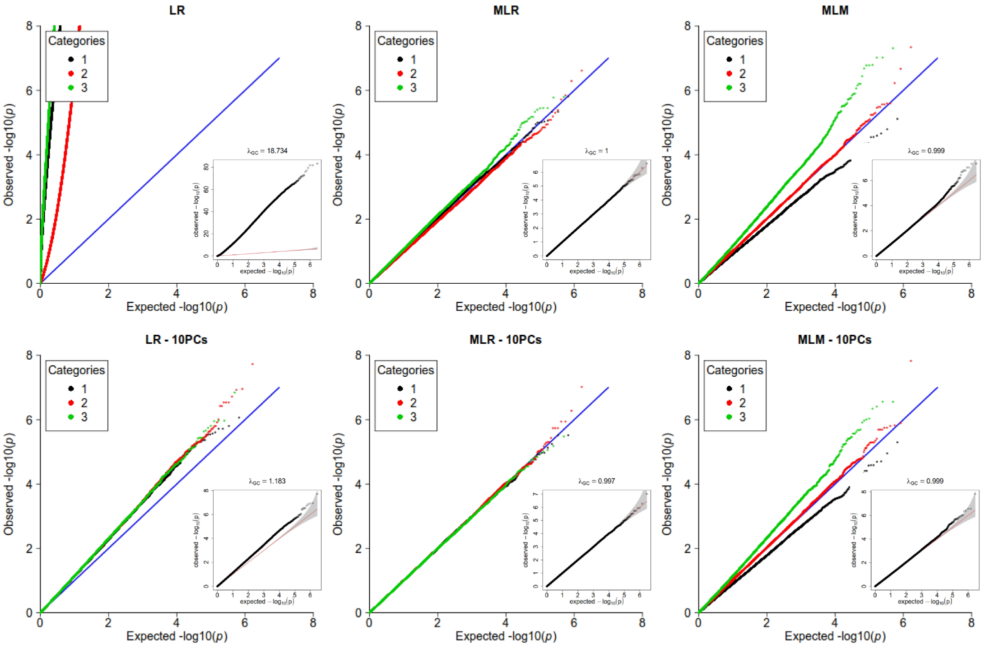

Figure 1: Stratified quantile-quantile plots for logistic regression (LR), mixed logistic regression (MLR) using Chen's score test (or AMLE) and mixed linear model (MLM) on the data simulated with the coalescence model. On the second row, 10 PCs were included as covariates. SNP categories are determined as in Chen et al. (2016), based on the allele frequencies in the strata.

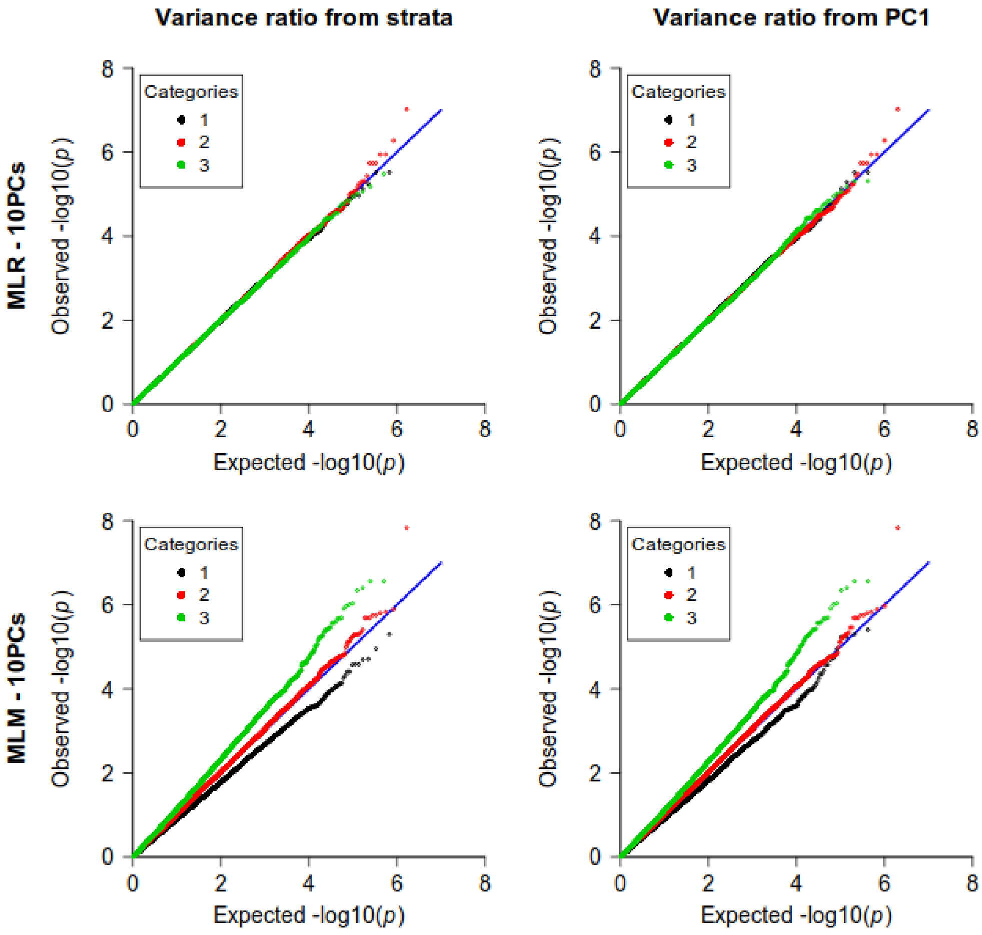

Figure 2: Stratified quantile-quantile plots obtained from the allele frequencies in the two strata (left) and from the first PC coordinates (right) for simulations based on the coalescence model.

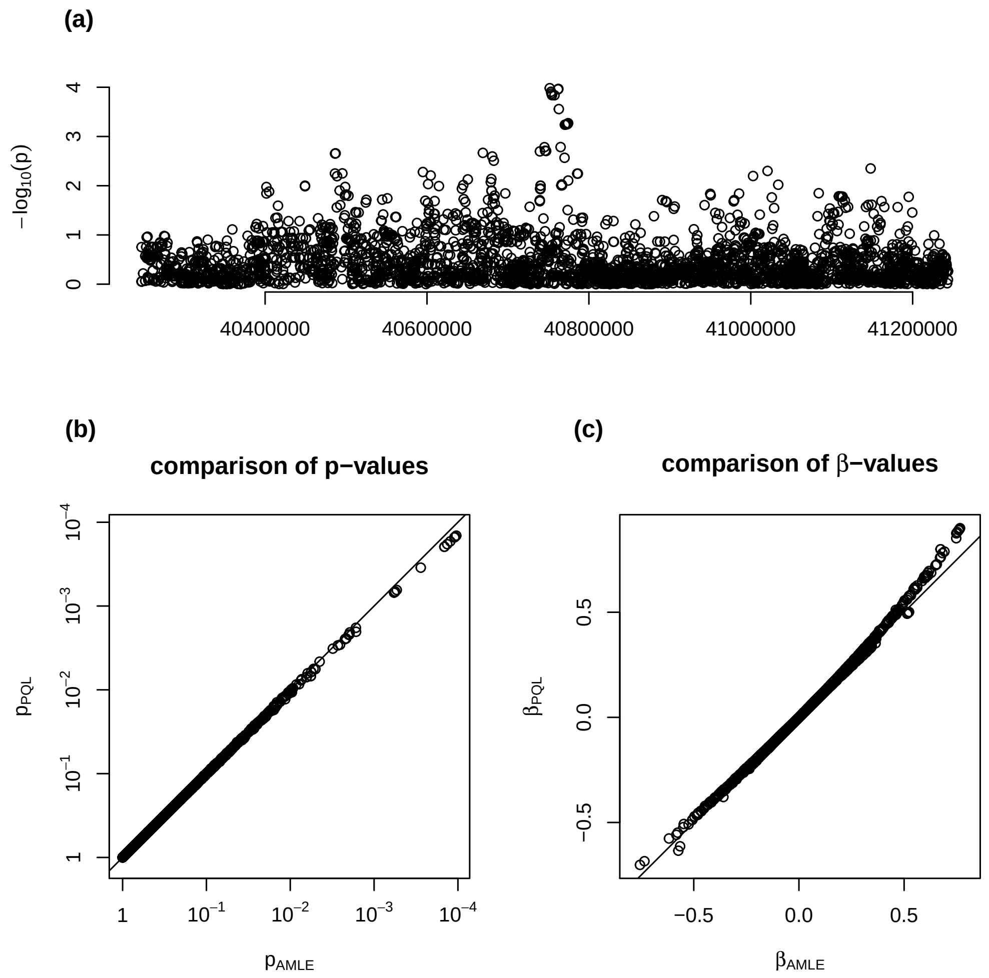

Figure 3: Association testing with Malaria infections, on a 100 kb segment on chromosome 20 (3461 SNPs with maf > 0.05). Panel (a): Manhattan plot. Panel (b): comparison of the p-values obtained by the AMLE and by the PQL. Panel (c): comparison of the β-values obtained by the AMLE and by the PQL.
